# Prevalence of Hypertension and Associated Factors among Residents Aged ≥18 Years in Ganzhou, China: A Cross-Sectional Study

**DOI:** 10.1155/2023/5486139

**Published:** 2023-10-05

**Authors:** Junwang Gu, Qi Wang, Wei Qiu, Fen Lin, Chunmei Wu, Ming Hao, Ping Wu

**Affiliations:** School of Public Health and Health Management, Gannan Medical University, Ganzhou 341000, Jiangxi, China

## Abstract

**Background:**

Reliable epidemiologic data on the present burden of hypertension are needed in developing region-specific strategies since previous studies have suggested that China is lagging in risk factor management.

**Objective:**

The study aimed at exploring the prevalence of hypertension and its associated factors among adult residents in Ganzhou, China. *Methodology*. A multistage, stratified, and cluster random sampling method was used to conduct the cross-sectional survey. The study selected four county-level districts in Ganzhou City, China, as the investigation area. Permanent residents aged ≥18 years who have settled in the area for 6 months or more were selected. A total of 7430 residents were involved in the study. Univariate and multivariate logistic regression analysis was carried out to identify predictors of hypertension.

**Results:**

The prevalence of hypertension in Ganzhou was 28.5%, and the age-standardized prevalence of hypertension was 22.4%. Univariate logistic regression analysis revealed that multiple variables have a statistically significant effect on the prevalence of hypertension. The elderly (odds ratio [95% CI]: 2.791 [2.594–3.004]), men (1.805 [1.583–2.058]), lower educated (0.848 [0.805–0.893]), a family history of hypertension (4.575 [3.900–5.368]), higher body mass index (1.344 [1.215–1.488]), central obesity (1.532 [1.304–1.799]), and less vegetable intake (1.150 [1.023–1.294]) were the major risk factors for hypertension.

**Conclusion:**

Hypertension is a major public health problem in Ganzhou and requires much effort to reduce its prevalence. An effective multifaceted implementation strategy is highly desirable to combat the emerging burden of hypertension.

## 1. Background

Hypertension, also regarded as high blood pressure, is a consistently elevated blood pressure disorder and a growing public health concern [1]. Hypertension increases the risk of heart, brain, kidney, and other disorders. It is the leading modifiable risk factor for cardiovascular diseases (including coronary heart disease, heart failure, stroke, myocardial infarction, atrial fibrillation, and peripheral artery disease) and premature death worldwide [1, 2]. Over a billion people worldwide, approximately 1 in 4 men and 1 in 5 women, have hypertension as of 2016 [3]. Two-thirds of hypertension are found in low- and middle-income nations, partly because there are more prevalent risk factors in those populations over the past few decades. Therefore, effective strategies focusing on the prevention and management of hypertension should be emphasized. Hypertension prevalence is regionally heterogeneous and may be partly explained by variations in the prevalence of risk factors for hypertension such as high sodium intake, low potassium intake, obesity, alcohol consumption, lack of physical activity, and unhealthy diet.

As the world's largest developing country, China's economic and social development is changing rapidly, which is accompanied by an altered lifestyle amongst its population. The prevalence of hypertension is remarkably heterogeneous among different populations associated with geographic, demographic, and socioeconomic characteristics [4–6]. Moreover, a concurrent change in epidemiologic characteristics may occur [5]. Ganzhou, a city in the Southern part of Jiangxi Province, China, has moderate economic development and significant variations in health determinants. The health level of its residents is a good representation of that of China. However, the current characteristics of hypertension and related risk factors among adult residents in Ganzhou, China, still need to be well studied. Therefore, the current study performed a cross-sectional survey of a large representative population in Ganzhou, China, to profile the characteristics of hypertension and corresponding risk factors for future research guidance on hypertension prevention and management strategies in China.

## 2. Methods

### 2.1. Study Area

**Figure 1:**
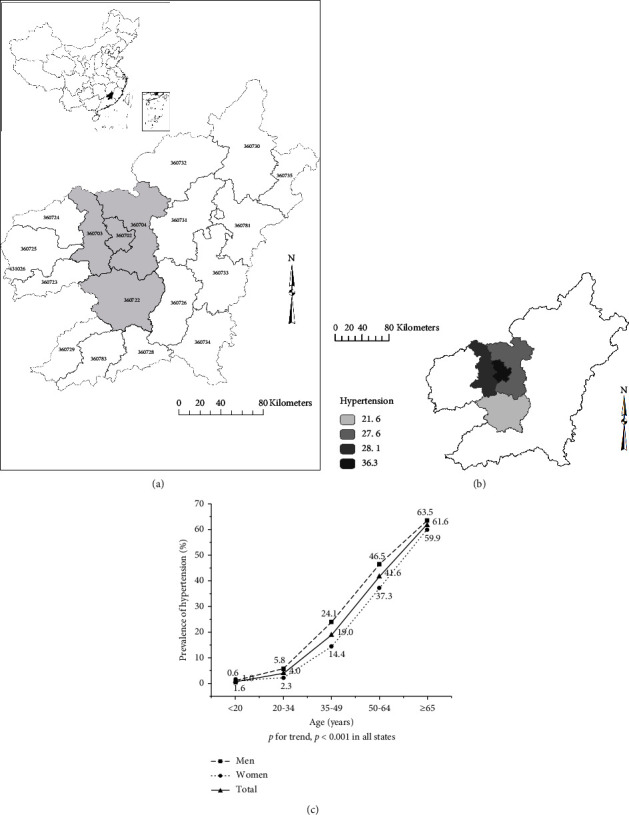
The prevalence of hypertension. *Note*. (a) The city of Ganzhou (black) and the four selected districts (gray) in this research; (b) prevalence of hypertension in four districts; (c) prevalence of hypertension in different age groups; ^*∗∗∗*^*p* < 0.001.

Ganzhou City (Figure 1(a)), with 18 county-level administrative districts, in the south of Jiangxi Province, China, is located between latitude 24°29′N and 27°09′N and between longitude 113°54′E and 116°38′E. The total area is 39379.64 km^2^, accounting for 23.6% of Jiangxi and making it the largest administrative region in Jiangxi Province. At the end of 2021, the city had a total registered population of 9.821 million.

### 2.2. Study Design and Participants

A multistage, stratified, and cluster random sampling method was used to conduct the cross-sectional survey in Ganzhou City, China, between 2018 and 2020. The study selected four county-level districts (Figure 1(a)): Zhanggong, Ganxian, Nankang, and Xinfeng districts in Ganzhou City as the investigation area. Permanent residents aged 18 years and above who have settled in the area for 6 months or more were selected.

Our research team partnered with the local Centers for Disease Control and Prevention (CDC) to study how social factors affect chronic diseases, and the data currently herein were only a portion of our overall findings. The survey was carried out under the guidance of the related County Health Commission and organized by the CDC in conjunction with the township health center in program design, on-site investigation, and residents' health examination.

### 2.3. Sampling Methodology

The sampling method was conducted in three stages: first stage: the townships in each district were divided into three strata according to their economic status, and two townships were randomly selected from each stratum; second stage: one or two administrative villages or resident committees were randomly selected from the selected townships; third stage: one or two villagers or resident groups were randomly selected from the selected administrative villages or resident committees, and then the research participants were randomly selected from the selected villagers or resident groups to do the investigation.

### 2.4. Sample Size

The formula mentioned below was adopted to calculate the sample size, referring to the average prevalence of chronic disease from 9.1% in the baseline year to 6.9% among rural residents across the five surveys, with the maximum prevalence of approximately 9.1% in Jiangxi Province as calculation base [7], with *α* = 0.05, *u*_*α*_ = 1.96, and *δ* = 15% *p*, to calculate the sample size of 1705 people.

(1) $$\begin{array}{c} n={\left(\frac{u_{a}}{\delta }\right)}^{2}p\left(1-p\right)\\ ={\left(\frac{1.96}{0.15\times 0.091}\right)}^{2}\times 0.091\times \left(1-0.091\right)\approx 1705. \end{array}$$

Considering that there may be a potential nonresponse rate, which we desired to be less than 10%, we distributed approximately 1870 questionnaires in each district. A total of 7430 valid questionnaires were collected, including 1830 in the Zhanggong district, 1868 in the Ganxian district, 1910 in the Nankang district, and 1822 in the Xinfeng county, respectively.

### 2.5. Tools of the Study

Residents' health investigation questionnaire primarily consists of basic resident information and behavioral risk factors questions such as diet, smoking, and alcohol intake. Part of the variables and variable groupings can be seen in Table 1. The standardized questionnaire was administered by the trained investigator who also measured the height, weight, and waist circumference of each participant in households according to the standard procedure.

### 2.6. Outcome Definitions

During the current study, participants were questioned about their medical history concerning hypertension. Those who reported being diagnosed with hypertension were classified as hypertensive, and their blood pressure was subsequently measured. Individuals who were found to have hypertension during the measurement were also included in the hypertensive group. (1) Prevalence of hypertension = (number of hypertension cases/number of respondents) × 100; (2) the systolic and diastolic blood pressure of the residents were measured three times consecutively and the average value was adopted as the reference for the diagnosis of hypertension; (3) the diagnostic criteria for hypertension are as follows: systolic blood pressure ≥140 mmHg and/or diastolic blood pressure ≥90 mmHg was considered hypertension [2]; (4) waist circumference ≥90 cm for men and ≥85 cm for women were the criteria for central obesity; and (5) passive smoking: the phenomenon of nonsmokers inhaling smoke produced by burning tobacco from the environment. It is passive smoking when the inhalation time is 15 minutes or more daily.

### 2.7. Statistical Analysis

EpiData (version 3.1, USA) was used to collect data and create the database. For statistical analysis, descriptive measures were performed using IBM SPSS (version 20.0, USA) software to summarize the principal results. The chi-square test was used for comparisons of categorical variables among groups. Student's *t*-test was performed to analyze continuous variables with a normal distribution. Odds ratios (ORs) with 95% confidence intervals (CIs) were calculated by univariate and multivariate logistic regression to evaluate the influencing factors of hypertension. Before conducting regression analysis, we preprocessed variables with 5–20% missing values. Two-sided *p* < 0.05 was statistically significant.

To compare with the national epidemiological data, the prevalence was age-standardized at the national level, using data from the 2010 Population Census of the People's Republic of China [8]. The ArcGIS 10.2 software was used to draw China maps and generate spatial distribution maps of the prevalence of hypertension.

## 3. Results

### 3.1. Characteristics of Respondents

The processing of missing values is described in Tables S1-S2 of the supplementary file, and the following statistics are all valid percentages with missing values excluded. As summarized in Table 1, there were 3571 men, accounting for 48.1% of the respondents, and 3859 women, accounting for 51.9%. Most respondents were Han Chinese, accounting for 99.1%. The average age of respondents was 47.28 ± 16.51 years, 46.86 ± 16.53 years for men, and 47.67 ± 16.48 years for women. Only 2.7% (201) of the respondents had an undergraduate or higher level of education. 2264 residents (30.6%) were “not working, such as jobless, retired.” Approximately 43.8% of the respondents had an average monthly income per capita of ≥1000 and <3000 yuan, and 32.8% had ≥3000 and <5000 yuan. In total, 1134 (38.4%) of the residents had a family history of hypertension. Smoking in Ganzhou City is 22.1% of the residents. Among nonsmokers, the proportion of passive smoking was 32.2% (1856/5772). Approximately 8.0% of the respondents have experienced being drunk. Daily consumption of vegetables by the respondents was 89.5%, 34.7% of the respondents had a daily fruit intake, and 46.0% consumed dairy products. Overall, 76.7% of the respondents slept for 6 to 8 h. The proportion of respondents who were overweight (body mass index (BMI) ≥24 and <28) and obese (BMI ≥ 28) was 23.9% and 6.1%, respectively, while 21.6% of respondents had central obesity.

### 3.2. The Prevalence of Hypertension

The prevalence of hypertension in Ganzhou was 28.5% (Table 1), and the age-standardized prevalence of hypertension was 22.4%. The prevalence of hypertension varied in different districts (*χ*^2^ = 98.536, *p* < 0.001). The closer to the city center, the higher the prevalence of hypertension, which went from 21.6% from the farthest point to the center and peaked at the Zhanggong district with a hypertension prevalence of 36.3% (Figure 1(b)). The prevalence of hypertension was higher in men (*p* < 0.001, Table 1). The prevalence of hypertension dramatically increased with age (*p* for trend <0.001, Figure 1(c)).

### 3.3. Associated Factors for Hypertension

As presented in Table 1, univariate analysis showed that multiple variables have a statistically significant effect on the prevalence of hypertension, besides the age and sex variables. More specifically, education, occupation, monthly average income per capita, family history of hypertension, the average amount of cigarettes smoked daily, frequency of getting drunk, and frequency of vegetable, fruit, and dairy products variables had a remarkable link with hypertension prevalence (all *p* values were <0.05).

In this survey, 7307 respondents provided information on their respective sleep time, and the average sleep time was 7.69 ± 1.29 hours. Mostly, respondents (76.7%) slept for between 6 and 8 h, 4.4% slept for less than 6 h, and 18.9% slept for more than 8 h.

The per capita daily salt intake (including salt in soy sauce and monosodium glutamate in the calculation) was 7.73 g. Overall, 58.0% of the residents in Ganzhou City exceeded the salt limit of 6 g per capita per day recommended by the Chinese Dietary Guidelines. There was no statistically significant difference in salt intake by sex (*t* = 1.129; *p* = 0.219). No statistically significant differences were found in the composition of salt consumption exceeding the limit by gender (*χ*^2^ = 0.075; *p* = 0.784) and by age group (*χ*^2^ = 9.393; *p* = 0.052). The salt consumption rate was slightly higher in hypertensive patients than in nonhypertensive (*t*′ = 2.036; *p* < 0.05).

The per capita daily intake of edible oil in Ganzhou City was 39.79 g (standard deviation 24.65 g), and only 10.9% of the respondents' average daily consumption of edible oil met the standard (25–30 g of oil per day recommended for adults in China), while up to 65.2% of the respondents' edible oil intake exceeded the standard. There was no statistically significant difference in the consumption of edible oil by sex (*t* = 0.515; *p* = 0.607).

Multivariate logistic regression analysis was performed on hypertension diagnosed in this survey and respondents without hypertension. Univariate analysis showed neither ethnic nor edible oil intake was statistically associated with hypertension. Furthermore, the multivariate analysis did not include occupation variables, such as “student” and “not working, such as jobless and retired,” due to their strong collinearity with age.

**Figure 2:**
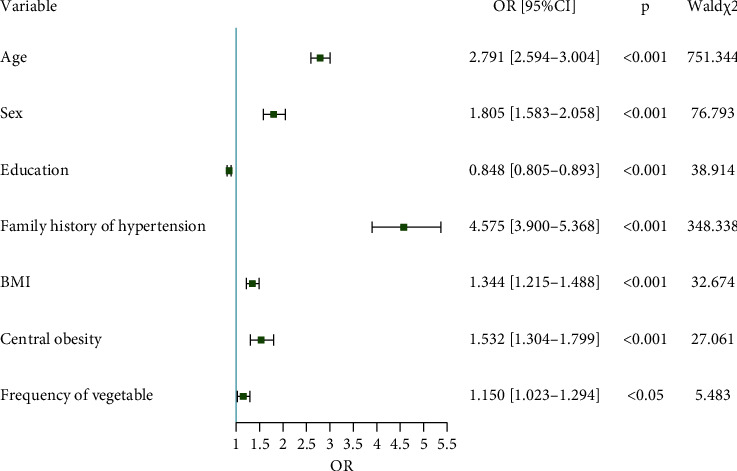
Multivariate logistic regression analysis.

Forward stepwise (likelihood ratio) with *α*_Enter_ = 0.05 and *α*_Remove_ = 0.10 was adopted to discover the principal risk factors for hypertension among Ganzhou residents. As presented in Figure 2, the elderly (OR [95% CI]: 2.791 [2.594–3.004]), men (OR [95% CI]: 1.805 [1.583–2.058]), lower degree of education (OR [95% CI]: 0.848 [0.805–0.893]), a family history of hypertension (OR [95% CI]: 4.575 [3.900–5.368]), higher body mass index (OR [95% CI]: 1.344 [1.215–1.488]), central obesity (OR [95% CI]: 1.532 [1.304–1.799]), and less vegetable intake (OR [95% CI]: 1.150 [1.023–1.294]) were the major factors for hypertension in Ganzhou.

## 4. Discussion

Hypertension is the most common and modifiable risk factor for increased cardiovascular morbidity and mortality in the population [2] and is the leading single contributor to all-cause death and disability worldwide [9]. Hypertension prevalence in China is increasing yearly with the changes in lifestyle, dietary structure, and the pressure of life in modern society [10, 11].

The current survey, from a large representative sample, provides new information on the status of prevalence and risk factors associated with hypertension among the adult population in Ganzhou. The results of this survey showed that the standardized hypertension prevalence in Ganzhou City was 22.4% for adult residents. These data are close to previous national surveys but higher than hypertension prevalence in nearby coastal regions such as Guangxi (15.8% from 2013 to 2015 [12]) and Shenzhen (19.2% as of 2018 [13]). Overall, the prevalence of hypertension among adult residents of Ganzhou is commensurate with the level of economic and social development. Ganzhou, as an ancient city that connects the less developed regions of Western China to the developed cities of the eastern coast, is undergoing profound changes in the social determinants of hypertension among its residents. Over the past decades, aging, urbanization, and associated unhealthy lifestyles and diets may explain the dramatic increase in the prevalence of hypertension [2, 14, 15]. Moreover, residents in developed areas tend to suffer from higher psychosocial stress, coupled with an individual unhealthy lifestyle, contributing to the development of hypertension [16].

The results showed geographical differences in the prevalence of hypertension among Ganzhou residents, which could be due to the variation in the geographical environment and economic development of the districts [6, 10]. Both univariate and multivariate logistic regression indicated that age and sex correlated with hypertension, which is consistent with previous findings [17]. With aging and physiological deterioration, for example, early vascular aging, the occurrence of various diseases (such as hypertension) inevitably increases [2, 18]. Compared to women, men had a much higher prevalence of hypertension, which might not only be due to sex, but also because men are more at risk for personal unhealthy behaviors associated with hypertension, such as smoking, physical inactivity, and alcohol consumption. Consistent with previous studies [19], family history is an important nonmodifiable risk factor for hypertension. The prevalence of hypertension was significantly higher in those with a family history of hypertension. Considering the growing trend of aging and the fact that 38.4% of residents have a family history of hypertension, the elderly, men, and those with a family history of hypertension should be a priority group for hypertension management.

Consistent with several studies [20], lower education is a primary risk factor for hypertension in residents of the Ganzhou district. This may be explained by (1) residents with lower education levels may be more deficient in health literacy, which has a very relevant role in health promotion. Studies have shown that inadequate health literacy levels were significantly associated with arterial hypertension, and being a male, having a low educational level, and being old were significant predictors of poor health literacy [21]; (2) some studies suggest that education level is not a causal risk factor for hypertension but rather serves as an indicator of socioeconomic status and a key determinant of hypertension prevalence [20]. Residents with undergraduate and higher education in Ganzhou were only 2.7%, suggesting that most residents have a low level of education. Therefore, in the short term, community health education to improve the health literacy of residents is essential for hypertension prevention, and over the longer term, the improvement of the education level of the whole population is a critical strategic practice for hypertension management.

Several literature studies suggest that overweight or obese people are more likely to develop hypertension, which is consistent with our findings. The proportion of respondents who were overweight and obese was 23.9% and 6.1%, respectively. Overall, 21.6% of the residents had central obesity. This is low compared with the 2017 national weighted prevalence of central obesity (35.4% [22]); however, given the close association between obesity and hypertension, residents must manage their weight properly.

A balanced diet is crucial to keep a healthy life and contributes to reducing the prevalence of hypertension [23]. This study found that the dietary intake of vegetables, fruits, and dairy products is associated with a lower prevalence of hypertension. Similar reports have been presented in previous studies [23–25] that the incident risk of hypertension was decreased by 1.9% for each serving per day of fruit consumption and decreased by 1.2% for each serving per day of total fruit and vegetable consumption [24]. A prospective epidemiological study from 21 countries showed that a higher intake of whole-fat (but not low-fat) dairy was associated with a lower incidence of hypertension [26]. Our results support the recommendation to increase the consumption of vegetables to prevent the risk of developing hypertension. Moreover, considering that lower salt intake correlated with better blood pressure control, keeping a low-salt diet is recommended for the prevention of hypertension, as shown by numerous literature findings [2, 23, 27].

Besides the factors mentioned above, univariate logistic regression analysis showed that multiple variables have a statistically significant effect on the prevalence of hypertension. Health disparities among different socioeconomic status groups remain a consistent finding in epidemiological studies [28]. Similar to education, occupation and income are indicators of socioeconomic status and are associated with the prevalence of hypertension [28, 29]. A previous study showed that unemployed or sedentary occupation workers reported being more susceptible than other occupation groups to cardiometabolic diseases when leisure time is spent being sedentary [30, 31]. Prior observational studies have revealed that hypertension is more common and more poorly controlled in lower socioeconomic groups [32]. The results showed a low income level with 43.8% and 32.8% of Ganzhou residents having an average monthly income per capita of ≥1000 and <3000 and ≥3000 and <5000 yuan, respectively. Priority actions are needed, especially in rural areas, to address the burden of hypertension in low- and middle-income populations.

Previous studies have shown [33–35] that unhealthy lifestyles such as smoking or passive smoking and alcohol consumption are risk factors for hypertension. Smoking has been widely reported to have a significant relationship with hypertension [33, 34]. A study has reported that light/moderate smoking or high cumulative smoking exposure accompanied by weight gain increased the risk of hypertension in Chinese men and smoking cessation decreased this effect [33]. Passive smoking has previously been demonstrated to be associated with increased prevalence and lower control of hypertension in a community-based elderly population [34]. The current survey found that 22.1% of the Ganzhou City residents smoke, and the proportion of passive smoking was 32.2% among nonsmokers. Health departments should conduct health education to reduce active smoking rates and take initiative to control environmental tobacco smoke exposure. This survey reported that approximately 8.0% of residents have experienced being drunk. Excessive alcohol consumption is a common adverse lifestyle. Population-based survey analysis showed an association between moderate and heavy alcohol consumption and a higher prevalence of systemic hypertension [36]. Curtailing alcohol consumption is the cornerstone in the management of hypertension owing to its significance as part of comprehensive lifestyle modifications [35]. Studies have shown that short sleep duration and poor sleep quality are associated with the prevalence of hypertension in the elderly [37]. The survey results showed that 4.4% of respondents slept less than 6 h and 18.9% slept more than 8 h. The prevention of hypertension in the elderly should be studied from the perspective of improving sleep quality [37].

## 5. Conclusion and Limitations

Altogether, hypertension is a major public health problem in Ganzhou, and much work remains to be done in an effort to reduce the prevalence of hypertension. The current study provides new information on the prevalence and risk factor profile of hypertension in the adult population of Ganzhou with a large representative sample. The factors influencing hypertension were consistent with the results reported in the previous literature. Taking appropriate precautions against these risk factors is a key aspect of hypertension management. Community outreach and long-term education strategies should be adopted to improve the health literacy of the residents. An effective multifaceted implementation strategy is highly desirable to combat the emerging burden of hypertension.

This study had some limitations. First, unlike prospective studies, the cross-sectional design prevents us from drawing causal and temporal inferences between potential risk factors and hypertension. Second, this study was conducted only in Ganzhou, which may affect its generalizability and representativeness due to regional differences.

## Figures and Tables

**Table 1:** Characteristics of respondents and the univariate logistic regression.

Variables	Grouping	Frequency (valid percent (%))	No. and prevalence rate (%) of hypertension	OR [95% CI]	*χ* ^2^	*p*
Age (years)	1 = “<20” (reference)	286 (3.8)	3 (1.0)	—	1510.371	<0.001
	2 = “20–34”	1566 (21.1)	63 (4.0)	3.954 [1.233–12.679]		
	3 = “35–49”	2315 (31.2)	439 (19.0)	22.075 [7.044–69.182]		
	4 = “50–64”	2021 (27.2)	841 (41.6)	67.232 [21.481–210.431]		
	5 = “≥65”	1242 (16.7)	765 (61.6)	151.289 [48.226–474.610]		

Sex	1 = “Men”	3571 (48.1)	1125 (31.6)	1.525 [1.361–1.709]	32.317	<0.001
	0 = “Women” (reference)	3859 (51.9)	986 (25.6)	—		

Ethnic	1 = “Hans”	7353 (99.1)	2090 (28.4)	1.127 [0.624–2.035]	0.032	0.857
	0 = “Others” (reference)	68 (0.9)	20 (29.4)	—		
	Missing value	9				

Education	1 = “No formal school education” (reference)	564 (7.6)	281 (49.8)	—	437.957	<0.001
	2 = “Did not finish primary school”	928 (12.6)	399 (43.0)	0.760 [0.616–0.937]		
	3 = “Primary school”	1412 (19.1)	491 (34.8)	0.537 [0.440–0.655]		
	4 = “Junior high school”	2739 (37.1)	560 (20.4)	0.259 [0.214–0.313]		
	5 = “Senior high school and technical secondary school”	1153 (15.6)	301 (26.1)	0.356 [0.288–0.439]		
	6 = “Junior college”	384 (5.2)	50 (13.0)	0.151 [0.107–0.212]		
	7 = “Undergraduate and above”	201 (2.7)	11 (5.5)	0.058 [0.031–0.109]		
	Missing value	49				

Occupation	1 = “Students” (other residents as a reference)	183 (2.5)	1 (0.5)	0.013 [0.002–0.096]	432.553	<0.001
	2 = “Institutional and enterprise staff” (other residents as a reference)	452 (6.1)	52 (11.5)	0.311 [0.232–0.417]		
	3 = “Scientific and technical staff” (other residents as a reference)	74 (1.0)	13 (17.6)	0.535 [0.293–0.976]		
	4 = “Other workers” (other residents as a reference)	4425 (59.8)	1052 (23.8)	0.574 [0.518–0.635]		
	5 = “Not working, such as jobless, retired, etc.” (other residents as a reference)	2264 (30.6)	981 (43.3)	2.747 [2.470–3.055]		

The monthly average income per capita (yuan)	1 = “<500” (reference)	288 (4.3)	92 (31.9)	—	28.621	<0.001
	2 = “≥500 and <1000”	552 (8.2)	205 (37.1)	1.259 [0.931–1.702]		
	3 = “≥1000 and <3000”	2950 (43.8)	821 (27.8)	0.822 [0.633–1.066]		
	4 = “≥3000 and <5000”	2207 (32.8)	580 (36.3)	0.759 [0.583–0.990]		
	5 = “≥5000”	736 (10.9)	198 (26.9)	0.784 [0.583–1.055]		
	Missing value	697				

Family history of hypertension?	1 = “Yes”	1134 (15.3)	684 (60.3)	5.362 [4.557–6.308]	669.778	<0.001
	0 = “No” (reference)	6296 (84.7)	1427 (22.7)	—		

The average amount of cigarettes smoked daily	1 = “Nonsmoker” (reference)	3916 (52.9)	1090 (27.8)	—	13.522	<0.05
	2 = “Nonsmoker with passive smoking”	1856 (25.0)	507 (27.3)	0.974 [0.861–1.103]		
	3 = “1–4”	341 (4.6)	111 (32.6)	1.251 [0.987–1.586]		
	4 = “5–9”	251 (3.4)	79 (31.5)	1.191 [0.904–1.568]		
	5 = “10–19”	617 (8.3)	175 (28.4)	1.027 [0.850–1.239]		
	6 = “20–29”	325 (4.4)	107 (32.9)	1.273 [0.999–1.620]		
	7 = “≥30”	100 (1.4)	38 (38.0)	1.589 [1.055–2.394]		
	Missing value	24				

Frequency of getting drunk	1 = “Almost daily (≥5 days per week)” (reference)	79 (1.2)	32 (40.5)	—	17.692	<0.01
	2 = “1–4 days per week”	93 (1.4)	29 (31.2)	0.666 [0.355–1.247]		
	3 = “1–3 days per month”	153 (2.2)	58 (37.9)	0.897 [0.515–1.563]		
	4 = “Less than 1 day per month”	288 (4.2)	100 (34.7)	0.781 [0.469–1.302]		
	5 = “Never”	6209 (91.0)	1751 (28.2)	0.577 [0.367–0.907]		
	Missing value	608				

Frequency of vegetable	1 = “Every day” (reference)	6577 (89.5)	1889 (28.7)	—	10.316	<0.05
	2 = “3–5 days per week”	532 (7.2)	130 (24.4)	0.803 [0.654–0.985]		
	3 = “1-2 days per week”	160 (2.2)	36 (22.5)	0.721 [0.495–1.048]		
	4 = “1–3 days per month”	32 (0.4)	8 (25.0)	0.827 [0.371–1.845]		
	5 = “Not eaten”	48 (0.7)	19 (39.6)	1.626 [0.910–2.907]		
	Missing value	81				

Frequency of fruit	1 = “Every day” (reference)	2544 (34.7)	688 (27.0)	—	43.868	<0.001
	2 = “3–5 days per week”	1824 (24.9)	488 (26.8)	0.985 [0.860–1.129]		
	3 = “1-2 days per week”	1672 (22.8)	438 (26.2)	0.958 [0.833–1.101]		
	4 = “1–3 days per month”	667 (9.0)	239 (36.1)	1.524 [1.272–1.827]		
	5 = “Not eaten”	621 (8.5)	211 (35.6)	1.490 [1.237–1.795]		
	Missing value	107				

Frequency of dairy products	1 = “Every day” (reference)	760 (10.3)	202 (26.6)	—	37.984	<0.001
	2 = “3–5 days per week”	843 (11.5)	194 (23.0)	0.826 [0.658–1.036]		
	3 = “1-2 days per week”	906 (12.3)	208 (23.0)	0.823 [0.658–1.029]		
	4 = “1–3 days per month”	876 (11.9)	271 (30.9)	1.237 [0.998–1.535]		
	5 = “Not eaten”	3972 (54.0)	1212 (30.5)	1.213 [1.019–1.444]		
	Missing value	73				

Sleep (hours)	1 = “<6” (reference)	319 (4.4)	164 (51.4)	—	87.831	<0.001
	2 = “6–8”	5604 (76.7)	1534 (27.4)	0.356 [0.284–0.447]		
	3 = “>8”	1384 (18.9)	371 (26.8)	0.346 [0.270–0.444]		
	Missing value	123				

BMI	1 = “<18.5” (reference)	503 (6.8)	97 (19.3)	—	218.805	<0.001
	2 = “≥18.5 and <24”	4678 (63.2)	1131 (24.2)	1.335 [1.059–1.682]		
	3 = “≥24 and <28”	1770 (23.9)	647 (36.6)	2.411 [1.894–3.071]		
	4 = “≥28”	455 (6.1)	225 (49.5)	4.095 [3.071–5.460]		
	Missing value	24				

Central obesity	1 = “Yes”	1590 (21.6)	688 (43.3)	2.361 [2.103–2.652]	217.602	<0.001
	0 = “No” (reference)	5779 (78.4)	1411 (24.4)	—		
	Missing value	61				

Hypertension	1 = “Yes”	2111 (28.5)				
	0 = “No”	5319 (71.5)				

## Data Availability

The data used to support the study are available upon reasonable request from the corresponding author.

## Supplementary Materials

Supplementary Materials Table S1: univariate Statistics of missing value analysis. Table S2: cross-tabulations of categorical versus indicator variables.
