# Supplementary material for: Prevalence of Hypertension and Associated Factors among Residents Aged ≥18 Years in Ganzhou, China: A Cross-Sectional Study
Source: Int J Hypertens. 2023 Oct 5;2023:5486139. doi: 10.1155/2023/5486139 (PMC10569894; doi:10.1155/2023/5486139)
Supplement: Supplementary Materials — Table S1: univariate Statistics of missing value analysis. Table S2: cross-tabulations of categorical versus indicator variables. [file 5486139.f1.pdf]

Processing of missing values

Missing values should be at most 20% of variables. Table S1 shows 1427 missing values (19.2%) for Salt intake. Monthly income and frequency of getting drunk are missing 697 (9.4%) and 608 (8.2%), respectively. Variables with less than 5% missing values won't be analyzed. We compared the rate of missing values for the three variables in hypertensive and non-hypertensive individuals and found no significant impact on outcome variables (Salt intake:18.9% vs. 20.0%; The monthly average income per capita:9.1% vs. 10.2%; Frequency of getting drunk: 8.8% vs. 6.7%), as shown in table S2.

Before conducting the regression analysis, we performed imputation separately for the three abovementioned variables due to missing values. The Replacement of missing values of the salt variable was processed by the method of Series mean substitution, while the other two were imputed with the method of the median of nearby points.

In summary, our larger sample size and pre-processing minimize the chances of significant bias in the results.

Table S1 Univariate Statistics of missing value analysis

Variables	N	Mean	Std. Deviation	Missing	
				Count	Percent
Salt intake (per capita daily)	6003	7.7264	14.05915	1427	19.2
Intake of edible oil (per capita daily)	7372	39.7949	24.65084	58	0.8
Age (years)	7430			0	0.0
Sex	7430			0	0.0
Ethnic	7421			9	0.1
Education	7381			49	0.7
Occupation	7398			32	0.4
The monthly average income per capita (Yuan)	6733			697	9.4
Family history of hypertension?	7430			0	0.0
The average amount of cigarettes smoked daily	7406			24	0.3
Frequency of getting drunk	6822			608	8.2

Frequency of vegetable	7349			81	1.1
Frequency of fruit	7323			107	1.4
Frequency of dairy products	7357			73	1.0
Sleep (hours)	7307			123	1.7
BMI	7406			24	0.3
Central obesity	7369			61	0.8
Hypertension	7430			0	0.0

Table S2 Crosstabulations of Categorical Versus Indicator Variables

			Total	Non-Hypertension	Hypertension
Salt intake (per capita daily)	Present	Count	6003	4314	1689
		Percent	80.8	81.1	80.0
	Missing	% SysMis	19.2	18.9	20.0
The monthly average income per capita (Yuan)	Present	Count	6733	4837	1896
		Percent	90.6	90.9	89.8
	Missing	% SysMis	9.4	9.1	10.2
Frequency of getting drunk	Present	Count	6822	4852	1970
		Percent	91.8	91.2	93.3
	Missing	% SysMis	8.2	8.8	6.7

Note: Indicator variables with less than 5% missing are not displayed.
